# Supplementary material for: Impact of storage conditions on the stability and biological efficacy of trans-arachidin-1 and trans-arachidin-3
Source: PLoS One. 2026 Jan 16;21(1):e0340979. doi: 10.1371/journal.pone.0340979 (PMC12810822; doi:10.1371/journal.pone.0340979)
Supplement: Attachment — Submitted filename: Response to reviewers.docx [file pone.0340979.s002.docx]

**Dear Editor,**

I am enclosing herewith a revised manuscript entitled “**Impact of storage conditions on the stability and biological efficacy of trans-arachidin-1 and trans-arachidin-3**” for publication in PLOS ONE for possible evaluation. The corresponding author of this manuscript is Apinun Limmongkon.

With this revision submission of the manuscript, I greatly appreciate the time and effort that you and the reviewers have dedicated to providing your valuable feedback on my manuscript. I have been able to incorporate changes to reflect most of the suggestions provided by the reviewers in this revised manuscript. The following is the response for the reviewers’ comment in each aspect.

All the yellow highlights in the revised manuscript are the corrected version which corresponded to the comments from reviewers.

**Reviewer #1: Manuscript ID: PONE-D-25-42817_reviewer**
Title: Impact of storage conditions on the stability and biological efficacy of trans-arachidin-1 and trans-arachidin-3
The paper focuses on the stability and biological efficacy of these compounds in both peanut hairy root culture crude extracts and partially purified fractions derived from elicited peanut hairy root cultures. The paper’s overall structure is coherent, with a logical flow. The content of this MS is abundant. However, some issues are suggested to be addressed before publication. See my comments below to improve this manuscript.

1. This work is limited to laboratory research and lacks performance comparison with existing emergency antioxidant products.

**Response:**

We sincerely appreciate the reviewer’s valuable comment. We agree that the present study primarily focuses on laboratory-scale evaluation of the stability and biological efficacy of trans-arachidin-1 (Ara-1) and trans-arachidin-3 (Ara-3) under different storage conditions. Our main objective was to establish fundamental data regarding the degradation and retention of these bioactive stilbenoids, which has not been previously reported. However, we acknowledge the importance of comparing the performance of these compounds with existing commercial antioxidant products to evaluate their potential practical applications.

We therefore added a statement in the revised Discussion (page 13, lines 446-449) to highlight this limitation as following statement: “Future studies should extend these findings by comparing the stability and antioxidant performance of Ara-1 and Ara-3 with established commercial antioxidant products under comparable storage and testing conditions to better assess their potential for practical applications.”

2. The quality of the pictures needs to be improved. The picture is blurry and indistinct.

**Response:**

All figures were reproduced in high resolution (≥ 1200 dpi) to ensure visual clarity for publication.

3. Which substances have antioxidant activity?

**Response:**

We thank the reviewer for this insightful question. A corresponding statement has been added to the Discussion section of revised manuscript (page 11, lines 381-385) to clarify this point as following statement: “The antioxidant activity observed in the peanut crude extract (PCE) is therefore likely attributed to these two predominant stilbenoids—Ara-1 and Ara-3—, which were identified and quantified as the major bioactive constituents. Consequently, their stability during storage was further monitored by HPLC quantification to evaluate their contribution to the overall antioxidant efficacy.”

4. The antioxidant activity is generally affected at high temperature, and what is the commercial value of the study at low temperature? Please provide more comprehensive evidence or references in introduction and/or discussion.

**Response:**

To address the reviewer’s concern, we have revised the Discussion sections (page 13, lines 439-443) to provide additional explanations regarding the relevance of low-temperature preservation as following statement “Low-temperature storage (4 °C to −20 °C) is commonly employed in commercial formulations of natural antioxidant products to minimize oxidative degradation and prolong product stability, as previously reported for polyphenolic-rich extracts and resveratrol derivatives [35]. Therefore, the present findings provide commercially relevant insights into the preservation of Ara-1 and Ara-3 under storage conditions.”

In summary, this is a well-designed and well-conducted study. The authors are encouraged to refine and improve the manuscript based on the above suggestions to showcase the value of the research better and lay a foundation for future studies.

**Reviewer #2: Comments**
The manuscript “Impact of storage conditions on the stability and biological efficacy of trans-arachidin-1 and trans-arachidin-3” is interesting and brings out the aspect of storage conditions affect the properties of stilbenes. There are only few things that can be addressed to make it clearer.

**Materials and methods**

1. The intext citations need to be properly aligned like…Pilaisangsuree, Somboon (12) …. And others

**Response:**

We thank for this helpful observation. We have carefully revised all in-text citations throughout the manuscript for properly aligned according to the journal’s reference style guidelines.

2. Line 96… half-strength might see inappropriate, indicate the concentration

**Response:**

In this study, half-strength (½ MS) refers to Murashige and Skoog medium containing 50% of the standard macronutrient and micronutrient concentrations. We have clarified this information in the revised manuscript (page 3, lines 94-96) as following statement: “Four grams of root tissue were cultured in 500 mL Erlenmeyer flasks containing 200 mL of half-strength Murashige and Skoog (MS) [13] liquid medium (2.2 g/L MS basal medium powder) supplemented with 1.5% sucrose.”

3. Line 104 can be rephrased to indicate the technique of separation…filtration or decantation

**Response:**

We have revised the sentence in the manuscript to specify the separation technique. The revised sentence is provided on (page 3, lines 103-105) as following statement: “The culture medium was subjected to liquid–liquid extraction using an equal volume (200 mL) of ethyl acetate, repeated three times, and the organic phase was separated by decantation to ensure efficient extraction.”

4. Line 107…. rotary evaporator (Büchi)… is not clear is it the type of evaporator?

**Response:**

We have revised the sentence in the Materials and Methods section (page 3, lines 105-107) to specify the exact type of the instrument for clarity as following statement: “The combined organic phases were concentrated under reduced pressure at 40 °C using a rotary evaporator (Büchi Rotavapor, Switzerland) to obtain the PCE for subsequent stability assays.”

5. Line 130….also the references, and also indicate the equipment for reading absorbance

**Response:**

We have carefully revised all in-text citations to align the journal’s reference style guidelines.

The microplate reader has been indicated in the revised manuscript (page 4, lines 124-125 and lines 133-134 ) as following: “using a microplate reader (Varioskan LUX, Thermo Scientific, USA)”

6. Line 143 …how did you determine the Fractions containing a predominant amount of Ara-1 and Ara-3

**Response:**

This clarification has been added to the revised manuscript (page 4, lines 143-144) as following statement:

“Fractions containing a predominant amount of Ara-1 and Ara-3 were identified by comparing their HPLC retention times with purified standards Ara-1 and Ara-3.”

7. Concentrations…200- 3200 could be better written as 0.2-3.2mg/mL

**Response:**

We thank the reviewer for the thoughtful suggestion. We agree that expressing concentrations in mg/mL is scientifically valid; however, in this study we intentionally presented all treatment concentrations in μg/mL to maintain consistency across experiments conducted with both SW480 and KKU-100 cell lines. Since the concentration range used for KKU-100 cells was 100–500 μg/mL, using the same unit facilitates direct comparison between the two cell models and ensures clarity in data interpretation. This rationale has been retained in the revised manuscript for consistency in reporting.

However, we included the following sentence to the revised manuscript (page 5, lines 170-172) to clarify this point: “All treatment concentrations were expressed in μg/mL to maintain consistency across experiments conducted with different cell lines and to facilitate direct comparison of cytotoxic responses.”

8. Why the different concentrations of the DMSO

**Response:**

The different DMSO concentrations resulted from the variation in the stock concentrations and the maximum test doses of the tested samples in each experiment.

- In the first experiment (using only PCE), the stock concentration was 400 mg/mL, and the maximum final concentration tested was 3200 µg/mL, resulting in a final vehicle concentration of 0.8% DMSO.
- In the second experiment (using PCE and partially purified fractions, Ara-1 and Ara-3), the stock concentration was 100 mg/mL, and the highest test concentration was 500 µg/mL, leading to a final vehicle concentration of 0.5% DMSO.

Thus, the DMSO level varied proportionally to the solubility requirement of each test sample while ensuring that the final DMSO concentration remained below 1%, which is non-toxic to the tested cells.

To improve clarity, the stock concentrations and corresponding vehicle concentrations have now been clearly stated in the Materials and Methods, section 2.8 Cell viability assay of the revised manuscript as following statement:

“The SW480 cells were continuously treated with the PCE (stock concentration at 400 mg/ mL)” (page 5, lines 173) of the revised manuscript.

“For KKU-100 cells, treatment included PCE, Ara-1 and Ara-3 (stock concentration at 100 mg/ mL)” (page 5, lines 176) of the revised manuscript.

9. …. GraphPad Prism software version 9.4.0… should be in the analysis section
**Response:**

The sentence: “GraphPad Prism software version 9.4.0…” has been moved to the analysis section (page 7, lines 251) of the revised manuscript.

**Results**
1. …This color change suggests…is well suited for the discussion part

**Response:**

We have moved the sentence “This color change suggests…” from the Results section to the Discussion section of revised manuscript (page 11, lines 377-381) as following statement: “After 72 hours of elicitation, the hairy root culture medium exhibited a noticeable yellowish coloration compared to its initial state at 0 hour. This visible change is consistent with previous reports indicating that the secretion of stilbenoids into the medium often correlates with medium coloration [6]. In this study, the color change suggests the enhanced production and extracellular release of Ara-1 and Ara-3 in response to elicitor stimulation.”

3. The major stilbene content is not clear

**Response:**

To improve clarity, we have revised the wording from “major stilbene content” to “The predominant stilbenoids content” throughout the manuscript. This terminology more precisely describes Ara-1 and Ara-3 as the principal stilbenoids identified and quantified in the extract.

4. …This additional peak may represent…could be place d in the discussion section

**Response:**

We have moved the sentence “This additional peak may represent…” from the Results section to the Discussion section of revised manuscript (page 12, lines 407-409) and rewritten as “The presence of minor new peaks in the HPLC chromatograms supports this degradation pathway. The result suggest that low-temperature storage is more effective in preserving the chemical stability of stilbenoids in PCE over extended periods.”

5. The result section indicates the viability assay were done for three months and is not captured in the methods section clearly

**Response:**

The treatment period in the viability assay of SW480 cells (treatment for 3-months period) and KKU-100 cells (treatment for 6-months period) has been included in the Materials and methods section (page 5, lines 174-175) as following statement: “….. for a period of 3 months, with a vehicle containing 0.8% DMSO used as the negative control. For KKU-100 cells, treatments were conducted for 6 months”

6. In HPLC section you have trans-resveratrol (Res) which was not captured in other parts alongside trans-arachidin-1 (Ara-1) and trans-arachidin-3 (Ara-3).

**Response:**

In our study, trans-resveratrol (Res) was included as a standard for comparison. However, trans-resveratrol was not detected, suggesting that its concentration was below the detection limit or absent in the elicited samples. This clarification has been added to the result section of the revised manuscript (page 7, lines 220–222) as following statement: “Although Res was included as a reference standard, it was not detected in either the crude extract or the partially purified fractions, indicating that its concentration was below the detectable level under the applied analytical conditions.”

**Discussion**
The discussion is well captured especially the photodegradation

**Response:**

We sincerely thank the reviewer for the positive feedback of our discussion, particularly regarding the photodegradation aspect.

**Reviewer #3:**

The article " Impact of storage conditions on the stability and biological efficacy of trans-arachidin-1 and trans-arachidin-3" reported robust and diverse experiments to support their claims. Please address the following technical issues in a revised version:

1. Figure 2 a,b is it possible to represent these three month data points in bar graphs with individual data points included. As of now it is difficult to understand the spread of data points and these line graphs are very tightly packed, and error bars overlapping. Something to highlight the difference would be great. Although I highly recommend for ABTS assay, representing data in a table consisting of activity, km, kcat and kcat/km for efficient and clear presentation.

**Response:**

We have replaced the line graphs in Figure 2a-b, 4a-b, 7a-c with bar graphs that include individual data points to enhance clarity and visualization of data distribution. Additionally, the original line graphs which are useful for illustrating the overall trend of activity across the time period have been retained in the Supplementary file (Figure S1, S3 and S10). A corresponding table summarizing the ABTS assay data has also been included in the Supplementary Table S1 to provide a clearer representation of the data.

2. Figure 3. My suggestion is similar for product formation. Is it possible to integrate the peaks and normalize with the amount loaded on the column? Also, I am requesting a positive control (standard) and negative control (media minus products) if possible. The chromatogram traces are not really meaningful for comparison. Also statistics for hplc assay is not clear.
**Response:**

We thank the reviewer for the constructive comment. Figure 3 has been revised to illustrate the chromatograms of peanut crude extract (PCE) under different storage conditions, showing additional peaks in samples stored at room temperature due to thermal degradation. Ara-1 and Ara-3 standards were included as positive controls, while the culture medium without elicitation (negative control) is shown in the Supplementary Figure S2 were integrated and normalized to the amount of sample loaded on the column, and the quantified levels of Ara-1 and Ara-3 in both crude extracts and partially purified fractions are presented in Figure 7. Statistical analyses of the HPLC data have been clarified and included in Figure 7.

3. Figure 4, Figure 6 what assay was performed for stability testing? How are these figures different, except purification? Can any test of purity be shown?
**Response:**

Figure 4 focuses on the stability of each predominant stilbenoid—Ara-1 and Ara-3—under different temperature conditions (RT, 4 ºC, and -20 ºC), illustrating their degradation profiles over time. In contrast, Figure 7 (New Figure from Figure 6) presents the stability of the partially purified fraction containing Ara-1 and Ara-3 stored at only −20 °C for 6 months. This analysis was performed to confirm the stability of these compounds prior to their use in the cell-viability assays, thereby ensuring that the observed biological effects were associated with stable and quantifiable levels of Ara-1 and Ara-3.

Regarding the reviewer’s question on purity, the partially purified fraction was verified by HPLC, Mass spectrometric analyses, and NMR analyses. The HPLC chromatograms showed distinct and well-resolved peaks corresponding to Ara-1 and Ara-3 when compared with in-house purified standards. The Mass and NMR data further confirmed the molecular identity and structural characterization of these compounds. The method of “2.7 Structural identification and characterization of Ara-1 and Ara-3” has been added to the Material and method section of the revised manuscript (page 5, lines 146-158). Additionally, the result of Ara-1 and Ara-3 characterization has been added to the Result section “3.3 Isolation and characterization of trans-arachidin-1 and trans-arachidin-3” of the revised manuscript (page 8-9, lines 267-300) with the new Figure 6 and Supplementary Figure S5-S9.

4. Figure 5. and Figure 7. A control/sham group seems to be missing (cell lines' data without treatment) for both cell type data? Please check to add any relevant data.

**Response:**

The untreated control data were indeed included in all experiments and were used as the baseline reference for calculating cell viability. As stated in the Materials and Methods (**section 2.8 Cell viability assay,** (page 5, lines 180-181) **:** “Cell viability was calculated as a percentage relative to the control group.”

Hence, the control group data are not absent but are integrated into the calculation of percentage cell viability and IC₅₀ values, which are presented in Figures 5 and 9 and the Supplementary Figures S4, S11.

**Reviewer #4:**

In this manuscript, the authors investigated the effects of three storage conditions on the biological stability and activity of Ara-1 and Ara-3 isolated from PCE. Stability and activity were assessed using HPLC analysis, cytotoxicity assays, and antioxidant assays. Overall, the study is of interest; however, several issues should be addressed:

1. The authors reported that PCE stored at room temperature (RT) exhibited a greater decline in antioxidant activity compared with samples stored at 4 °C and –20 °C. However, a gradual decrease in antioxidant activity was also observed even at –20 °C. The authors should discuss possible reasons for this loss and propose strategies to minimize it.

**Response:**

The additional discussion of this aspect has been included in the Discussion section of the revised manuscript (page 11, lines 397-401) as following statement: “Although a gradual decrease in antioxidant activity was observed at −20 °C, the rate of decline was significantly lower than that at room temperature, indicating greater stability under low-temperature storage. The minor loss of activity may be attributed to slow oxidative degradation or light-induced structural alterations occurring during prolonged storage [30].”

2. In Figure 4b, samples stored at RT showed a marked reduction in Ara-3 content. The authors should clarify what degradation products are formed and describe the potential chemical mechanism underlying this process. Relevant reaction pathways or schemes would strengthen this discussion.

**Response:**

We have now included a description of the proposed mechanism of the possible photochemical and oxidative degradation pathways in the Discussion section of the revised manuscript (page 12, lines 403-407) as following statement: “The pronounced reduction in Ara-1 and Ara-3 content observed under room temperature storage is likely due to photo-oxidation and thermal degradation, processes commonly reported for stilbene compounds [31]. Exposure to light and oxygen can induce trans–cis isomerization of the stilbene double bond, followed by oxidative cleavage or polymerization, resulting in the formation of less active phenolic derivatives [32].”

3. In Section 3.3.1, the authors present the stability of purified Ara-1 and Ara-3 over time under different conditions. What changes were observed in their antioxidant activities during storage?

**Response:**

To address this point, the antioxidant activity of the PCE, Ara-1, and Ara-3 evaluated at 0 and 6 months was included in the result section “3.4.2 Stability of **in vitro antioxidant activity** and cytotoxic activity of KKU-100 cholangiocarcinoma cells” of the revised manuscript (page 10, lines 338-346) as following statement: “The *in-vitro* antioxidant activity of PCE, Ara-1, and Ara-3 was determined at 0 and 6 months of storage using the ABTS assay, and the results are presented in Fig 8 and summarized in Supplementary Table S4. A significant reduction in TEAC values was observed after 6 months for all samples (p < 0.05), with activity decreasing by approximately 1.2- to 1.3-fold compared with the initial levels. Among the samples, Ara-1 and Ara-3 exhibited higher antioxidant capacities than the crude extract, both at the beginning and after storage, indicating that these predominant stilbenoids contribute substantially to the antioxidant potential of the PCE and remain relatively stable even after long-term storage under low-temperature conditions.”

4. In Figure 7, PCE treatment significantly increased cell viability at 100 µg/mL. The authors should provide an explanation or hypothesis for this observation.

**Response:**

We thank the reviewer for this insightful comment. Previous studies have described similar low-dose stimulatory effects for stilbenoids such as resveratrol and arachidin derivatives, which can enhance cell survival at low concentrations, but induce apoptosis at higher doses. These mechanistic insights have now been briefly discussed in the Discussion section of the revised manuscript (page 12, lines 428-436), with the relevant references incorporated as following statement: “Interestingly, a slight increase in cell viability was observed at the lower concentration (100 µg/mL) of PCE compared with the untreated control. This biphasic, or hormetic, effect has been widely reported for polyphenolic compounds, where low concentrations stimulate cell proliferation or activate antioxidant defense mechanisms, while higher concentrations exert cytotoxic or pro-apoptotic effects. The mild stimulatory response observed here may result from minor bioactive constituents within the crude extract that exert cytoprotective or antioxidant effects at sub-toxic levels. Similar dose-dependent responses have been reported for stilbenoids such as resveratrol and arachidin derivatives, which can activate the Nrf2/ARE-mediated antioxidant pathway, enhancing cellular defense and survival at low concentrations but inducing apoptosis at higher doses [4, 21].”

5. The statistical analyses across figures are insufficiently described. Many figures lack p-value information, and the criteria for statistical significance are unclear. For instance, in Figure 7a, all three groups show significant differences at several concentrations—were these comparisons made versus untreated controls or between treatment groups?

**Response:**

For the antioxidant assay and stilbene content analysis, statistical comparisons were conducted among different storage periods within each storage condition to evaluate temporal stability. In contrast, for the cell viability assay, statistical comparisons were made relative to the untreated control group rather than between treatment groups to assess treatment effects.

To address the reviewer’s comment, the Materials and Methods section (page 6, lines 187) has been revised to clearly define these comparison criteria and significance thresholds as follow: “statistical significance set at p < 0.05, 0.01, 0.001..” Figure legends (Figure 9 and Supplementary Figures S4, S11) have also been updated to clarify that asterisks indicate the level of significance for each comparison type.
