# Supplementary material for: Impact of storage conditions on the stability and biological efficacy of trans-arachidin-1 and trans-arachidin-3
Source: PLoS One. 2026 Jan 16;21(1):e0340979. doi: 10.1371/journal.pone.0340979 (PMC12810822; doi:10.1371/journal.pone.0340979)
Supplement: Attachment — Submitted filename: Response to reviewers2.docx [file pone.0340979.s003.docx]

**Dear Editor,**

I am enclosing herewith a revised manuscript entitled “**Impact of storage conditions on the stability and biological efficacy of trans-arachidin-1 and trans-arachidin-3**” for publication in PLOS ONE for possible evaluation. The corresponding author of this manuscript is Apinun Limmongkon.

With this revision submission of the manuscript, I greatly appreciate the time and effort that you and the reviewers have dedicated to providing your valuable feedback on my manuscript. I have been able to incorporate changes to reflect most of the suggestions provided by the reviewers in this revised manuscript. The following is the response for the reviewers’ comment in each aspect.

All the yellow highlights in the revised manuscript are the corrected version which corresponded to the comments from reviewers.

**Reviewer #3:**

I thank the authors for their kind consideration of the reviewer's comments. The manuscript is definitely shaping up better than previous submission. But presentation is not at par with publication standards. I urge the authors once again, to consider the following suggestions. Strictly aimed to concise redundant information and consider improving the figures with more traditional approaches for better understanding.

**1. HPLC Y Axis units for PDA detector should be mAU? Please check.**

**Response:**

We thank the reviewer for the helpful comment. In our HPLC system, the PDA detector records the chromatographic signal as an analog voltage output (V), which is proportional to UV absorbance. Therefore, the Y-axis was originally presented in volts. To avoid confusion, we have now clarified this point in the Methods section (Page 4, Line 121-123) and revised the Figure 3 captions to explicitly indicate the detector response units.

**2. Line 210: Where is table 1 in main text? Is it SI Table 1?**

**Response:**

We thank the reviewer for highlighting this issue and apologize for any confusion caused. Table 1 is supplied as a separate standalone file, uploaded independently of the Supplementary Information and also include in the main text.

**3. Line 225-227: "The freshly prepared PCE at 0 month (initial time point) (Figure 3c), together with samples stored for 3 months at RT (Figure 3d), 4 °C (Figure 3e), and −20 °C (Figure 3f), exhibited a gradual decline in the peak intensities of Ara-1 and Ara-3 under all storage conditions, indicating time-dependent degradation of these stilbenoid compounds". I still hold my reservation with this claim.** **The peaks were not integrated to calculate intensities. Is table S2 data relevant to this peak intensity calculation? If not, kindly provide. A LC chromatogram merely confirms time of retention and since extraction from replicates using organic solvents would be different, I expect a large variation in HPLC input samples. Normalizing against per gram crude extract may not suffice.**

**Response:**

We thank the reviewer for this thoughtful comment. We would like to clarify that the chromatograms presented in Figure 3 were subjected to peak integration, and the resulting peak areas were used to quantify the two predominant stilbenoids, arachidin-1 (Ara-1) and arachidin-3 (Ara-3), at each storage temperature. The quantified results are presented in Figure 4 and summarized in Table 1 and Supplementary Table S2. A detailed description and interpretation of the quantitative HPLC data have been described in the manuscript (page 8-9, lines 250–266), as highlighted in the revised version.

With respect to the variation observed in the quantified HPLC results, we would like to clarify that the primary objective of this experiment was to evaluate the stability of the crude extract under different storage temperatures, rather than to assess variability arising from independent extraction procedures. To this end, a single batch (lot) of crude extract was prepared, thoroughly homogenized, and subsequently aliquoted into independent triplicates, which were then stored at −20 °C, 4 °C, and room temperature (RT) for a period of three months. Because all samples originated from the same extraction lot and were subjected to identical handling prior to storage, the observed variation in HPLC peak areas among replicates was minimal. This low variability therefore reflects the controlled experimental design and is appropriate for isolating the effect of storage temperature on extract stability. We agree that normalization to crude extract mass may not fully account for variability arising from independent extractions; however, in this study, the use of a single extraction batch was intentional in order to specifically evaluate temperature-dependent stability effects. This clarification has now been added to the Material and Method section (page 3-4, lines 107-111 and page 5, lines 150-155) and the Discussion section (page 12, lines 384-391) of the revised manuscript.

**4. Line 240: i am again confused how Figure 4 and Table 1/S1 are related?**

**Response:**

We thank the reviewer for raising this point and apologize for any confusion. The quantified amounts of arachidin-1 (Ara-1) and arachidin-3 (Ara-3) in the crude extract, obtained from integrated HPLC peak areas, are graphically presented in Figure. 4 to facilitate visual comparison among storage conditions. The corresponding numerical values (mean ± SD) derived from independent replicates are summarized in Table 1.

In accordance with PLOS ONE data-reporting requirements, which request disclosure of

(i) the values underlying reported means and standard deviations

(ii) the values used to generate figures

Therefore, the raw quantitative data from each independent replicate have been provided in Supplementary Table S2. This table contains the individual data points used to calculate the mean ± SD values presented in Fig. 4 and Table 1.

**5. Figure 5: Can you kindly present this figure in a different way like a typical IC50 curve? At the moment it is incomprehensible. Instead of merely putting the IC50 numbers, please use %viability on Y and conc of compound on X and make 3 plots for RT, 4 and -20. The reason I suggest this is again, because the line curves are too close to each other for significance, unless your data is extremely reproducible, these are difficult to comprehend.**

**Response:**

We thank the reviewer for this valuable suggestion regarding data presentation and agree that conventional concentration–response curves (% cell viability versus concentration) provide clearer visualization of cytotoxic activity.

In response to the reviewer’s comment, and following discussion within the research team, we have revised Figure 5 to adopt the same graphical presentation style as Figure 8. Figure 5 now presents concentration–response (% cell viability) curves of peanut hairy root culture crude extract (PCE) against SW480 colon cancer cells at each storage temperature (RT, 4 °C, and −20 °C) and time point (0–3 months). In this revised figure, panels (a–c) display concentration–response effects expressed as percentage cell viability compared with the vehicle control, while panel (d) summarizes the corresponding IC₅₀ values derived from these curves.

To improve manuscript flow, clarity, and statistical transparency, we further clarified that different statistical comparisons were applied to different panels: concentration–response curves (Figure 5a–c) were analyzed relative to the vehicle control, whereas IC₅₀ values (Figure 5d) were statistically compared with the corresponding initial time point (0 month) within each storage condition. This approach allows direct visualization of cytotoxic effects while maintaining IC₅₀ values as a quantitative measure of storage-dependent bioactivity changes.

To ensure consistency throughout the manuscript, we revised multiple sections as detailed below. This revised presentation ensures that cytotoxicity data in Figure 5 are directly comparable in style and interpretation to those shown in Figure 8, thereby facilitating clearer understanding for readers.

**Summary of revisions made in the manuscript**

1. **Figure 5:**

Reconstructed to present concentration–response (% cell viability versus concentration) curves for PCE stored at RT, 4 °C, and −20 °C over 0–3 months (panels a–c), with statistical comparisons performed relative to the vehicle control. Panel (d) summarizes the corresponding IC₅₀ values derived from these curves, with statistical comparisons made against the initial time point (0 month).

2. **Figure 5 legend:**

Revised to clearly define the experimental scope and statistical comparisons applied to each panel, specifying that panels (a–c) were analyzed relative to the vehicle control, while panel (d) was analyzed relative to the initial time point.

3. **Results section (Section 3.2.3):**

Revised to describe cytotoxicity trends based on concentration–response curves, explicitly distinguishing between vehicle control–based comparisons for dose–response data and time point–based comparisons for IC₅₀ values (page 9, lines 269-293).

4. **Supplementary Information Figure S4:**

Updated cross-references to ensure consistency between Figure 5, the corresponding IC₅₀ dataset, and the revised statistical descriptions in the Results section.

**6. Results Section 3.3 - peak shift information are not needed in main text. Kindly consider omitting. In fact, please consider revision of this entire section and making the figure more informative. I cannot comprehend how this result section fits in your manuscript flow. Where is control data for NMR and also negative control?**

**Response:**

We thank the reviewer for this comment. As suggested, Section 3.3 has been relocated to the Supplementary Information (Supplementary Figs. S5–S10). The corresponding results statement referring to the NMR and HRMS analyses has been rewritten in the main text (page 10, lines 299–302).

With regard to the control data for NMR spectroscopy, we would like to clarify that structural validation was achieved through analysis of chemical shifts, coupling patterns, and signal multiplicities of Ara-1 and Ara-3, in comparison with previously reported reference data from the literature. The relevant literature references have now been incorporated into the caption of Supplementary Fig. S10. Chemical shifts were referenced to residual solvent signals, and the structural assignments were further supported by complementary HRMS analysis.

**7. Figure 7: Exactly what tests are performed here? Kindly update all legends with as much information as possible. When a reviewer is evaluating this manuscript it is the figure legends that help in understanding the figure. Going back and forth to the methods section is really an unfeasible approach.**

**Response:**

We thank the reviewer for raising this important point and apologize for the lack of clarity in the original figure legend. As some figure was moved to the Supplementary Information, the former Figure 7 has been renumbered as Figure 6.

Figure 6 presents the quantification and stability of the predominant stilbenoids in (a) peanut hairy root culture crude extract (PCE), (b) partially purified fraction enriched in trans-arachidin-1 (Ara-1), and (c) partially purified fraction enriched in trans-arachidin-3 (Ara-3) during storage at −20 °C over a six-month period. Stilbenoid contents were quantified by HPLC–UV analysis based on integrated peak areas and calculated using authenticated Ara-1 and Ara-3 reference standards. All samples were derived from a single purification batch, aliquoted, and stored at the indicated time points prior to analysis. The revised and expanded figure legend has been added to the caption of Figure 6.

**8. Section 3.4.2 and Section 3.2.3 - why two different cell lines were chosen? Do you have any viability data related to Fig 5 for KKU-100 line? For consistency, can the cell data be considered to be represented together? The Figure 9 viability assay is well done and my suggestion was the same for Figure 5. Kindly consider a better flow of data for better understanding for readership.**

**Response:**

We appreciate the reviewer’s thoughtful comment regarding cell line selection and data consistency. As some topic was moved to the Supplementary, the former Section 3.4.2 has been renumbered as 3.3.2. As some figure was moved to the Supplementary Information, the former Figure 9 has been renumbered as Figure 8.

The use of two different cancer cell lines was intentional and aligned with the specific objectives of each experimental section. SW480 colon cancer cells (Section 3.2.3, Figure 5) were used to evaluate the short-term (3-month) stability of the crude extract (PCE) under different storage temperatures. SW480 cells exhibit moderate sensitivity to polyphenol-rich crude extracts, allowing detection of gradual changes in cytotoxic activity during storage.

In contrast, KKU-100 cholangiocarcinoma cells (Section 3.3.2, Figure 8) were used to assess the long-term (6-month) stability of partially purified Ara-1 and Ara-3 fractions, as these cells are more sensitive to arachidin compounds, enabling clearer discrimination of bioactivity changes over prolonged storage.

Viability data of PCE against KKU-100 cells are included in Figure 8 and Supplementary Figure S12, where PCE is presented alongside Ara-1 and Ara-3 at both 0 and 6 months. These results demonstrate that PCE retains relatively stable cytotoxic activity in KKU-100 cells, supporting consistency between crude and purified samples.

To improve manuscript flow, clarity, and figure consistency, we revised multiple sections of the manuscript as detailed below. This stepwise experimental design demonstrates bioactivity stability across different cancer models and extract complexities without unnecessary duplication of experiments.

**Summary of revisions made in the manuscript**

1. **Methods section (Section 2.8, Cell viability assay):**
   Added a clear rationale for cell line selection, specifying that SW480 cells were used to assess short-term cytotoxic stability of the crude extract, whereas KKU-100 cells were selected for long-term evaluation of partially purified arachidin fractions due to their higher sensitivity (page 6, lines 192-195).
2. **Results section (Section 3.2.3):**
   Clarified that cytotoxicity data in SW480 cells represent short-term (3-month) stability of the crude extract and added a forward reference to Section 3.3.2 to indicate subsequent evaluation in a more sensitive cancer model (KKU-100 cells) (page 9, lines 269-293).
3. **Results section (Section 3.3.2):**
   Explicitly stated that the crude extract (PCE) was also evaluated in KKU-100 cells at 0 and 6 months, enabling direct comparison between crude and purified samples under identical experimental conditions (page 11, lines 342-346).
4. **Figure legends (Figures 5, 8, and Supplementary Figure S12):**
   Revised the figure legends to clearly define the experimental scope of each figure, to specify the use of concentration–response (% cell viability) analyses, and to emphasize the complementary roles of Figure 5 (short-term stability of PCE in SW480 cells) and Figure 8 (long-term stability of PCE and purified fractions in KKU-100 cells). The legend of Supplementary Figure S12 was additionally revised to ensure consistency in data presentation and statistical annotation.
5. **Discussion section:**
   Added statement highlighting that, although different cancer cell lines were employed, consistent cytotoxicity trends were observed across models, reinforcing the robustness of the storage-dependent bioactivity findings (page 13, lines 434-436).
